# Distributed Key Management to Secure IoT Wireless Sensor Networks in Smart-Agro

**DOI:** 10.3390/s20082242

**Published:** 2020-04-15

**Authors:** Safwan Mawlood Hussein, Juan Antonio López Ramos, José Antonio Álvarez Bermejo

**Affiliations:** 1 Department of Computer Engineering, Faculty of Engineering, Tishk International University, Erbil 44001, Iraq; safwan.mawlud@tiu.edu.iq; 2 Department of Mathematics, University of Almería, 04120 Almería, Spain; 3 Department of Informatics, University of Almería, 04120 Almería, Spain

**Keywords:** elliptic curves, Diffie–Hellman, discrete logarithm problem, security protocol, secure key exchange, wireless sensor networks, sensor networks security, IoT networks, IoT security, smart-agro

## Abstract

With the deepening of the research and development in the field of embedded devices, the paradigm of the Internet of things (IoT) is gaining momentum. Its technology’s widespread applications increasing the number of connected devices constantly. IoT is built on sensor networks, which are enabling a new variety of solutions for applications in several fields (health, industry, defense, agrifood and agro sectors, etc.). Wireless communications are indispensable for taking full advantage of sensor networks but implies new requirements in the security and privacy of communications. Security in wireless sensor networks (WSNs) is a major challenge for extending IoT applications, in particular those related to the smart-agro. Moreover, limitations on processing capabilities of sensor nodes, and power consumption have made the encryption techniques devised for conventional networks not feasible. In such scenario, symmetric-key ciphers are preferred for key management in WSN; key distribution is therefore an issue. In this work, we provide a concrete implementation of a novel scalable group distributed key management method and a protocol for securing communications in IoT systems used in the smart agro sector, based on elliptic curve cryptography, to ensure that information exchange between layers of the IoT framework is not affected by sensor faults or intentional attacks. In this sense, each sensor node executes an initial key agreement, which is done through every member’s public information in just two rounds and uses some authenticating information that avoids external intrusions. Further rekeying operations require just a single message and provide backward and forward security.

## 1. Introduction

Agriculture has gone from being a major asset in the world’s food to being a key not only to food but also to sustainability and non-harmfulness, both to the environment and to people. The necessary increase in productivity to cope with the increase of the world’s population and the constraints under which this increase in productivity is subject due to climate change means that ecological procedures are not sufficient. It is in this context that the Internet of Things (IoT) emerges, as it has the potential to take control and monitoring (and thus decision-making) to another level [1].

**Figure 1:**
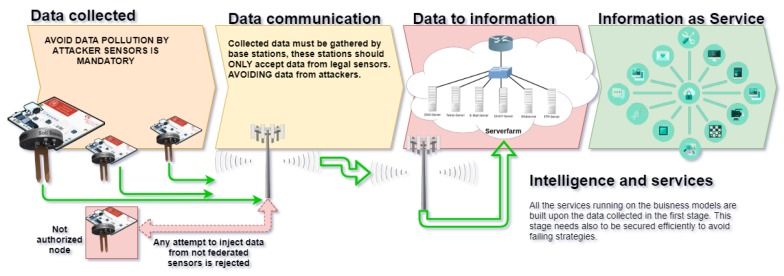
Stages from collecting data to processing information: the importance of securing the data.

The agricultural sector is facing impressive challenges arising from population growth, which demands more and more resources and their search has to be carried out in a sustainable manner. The use of technology is a crucial factor in achieving sustainable production. For instance, in [2], the authors set out the problem arising from the need to use water in a sustainable and responsible manner, which requires highly accurate and expensive sensor-based systems and IoT technology. However, manufacturers invest great efforts in generating accessible technology based on low-cost devices that are supported or required by data connections with nodes that allow realistic monitoring and therefore water savings. All of this is due to the advances in IoT and Wireless Sensor Network (WSN) technology, core to IoT that consists of tiny autonomous low-cost low-power devices that carry out monitoring tasks. WSNs can nowadays be found in many civil applications; see Figure 1. The devices, in Figure 1, in stage 1 (data collected) are called sensor nodes and the monitored data are typically sent to a base station, stage 2, that will be later processed in data mining servers, stage 3, to later serve as intelligence information used in stage 4 to adopt strategies. All of the sequence depends on the data gathered during stage 1. It is key to ensure that sensor nodes are operating properly, and that they are not being attacked or supplanted. Under these circumstances, the data gathered can drive the whole system to a failure, affecting the sustainability of the production. To this end, the proposed method secures the information exchange by means of a lightweight and efficient protocol implemented in each node. A faulty node (not callibrated) can request to be excluded from the secured group, avoiding that corrupted data reaches upper layers. On the other side, if new sources of information are intentionally created, these will be discarded as they are not part of the secured group of sensor nodes.

The evolution of technology has made it possible to create such integrated devices that, although limited in computational resources, can execute relatively complex tasks. This, together with the new wireless communication protocols specially designed for the exchange of information, allows the agricultural sector to equip itself with relatively cheap monitoring systems, capable of feeding information to decision support systems that allow decisions to be made about crop strategies and also allow resources to be saved. In [3], the authors demonstrate how precision agriculture and smart agriculture have evolved positively thanks to the application of these technologies. In addition, the authors of both [2,3] stress the need for security measures to ensure the quality of the data collected, as shown in Figure 1.

The work presented in this paper is a method to secure the information sent from sensors, in stage 1 (see Figure 1, to the Business Intelligence layer of the IoT Smart-Agro architectures. From its very beginning, the IoT architecture has been considered to be divided into three layers: the perception, network, and application layer. An evolution of this approach suggests that an intermediate layer is placed between the network and application layers, called a service layer. This service layer is submitted to sensors’ malfunctioning or by intentional attacks from, for example, competing companies (It is very easy to inject fake sensor data from soil sensors to the cloud, trying to emulate a valid sensor. This data can pollute how the—for example—water irrigation system will act when sending actuators the order to water olive-trees, for example). If fake information is sent to the gateway node, this information will be uploaded to the cloud. In addition, the result will produce an excessive watering of some trees that later will produce bigger and less productive olives (spending a lot of water in regions where the water scarcity is a problem), or, by not sending the correct amount of water, will negatively affect the tree.

**Figure 2:**
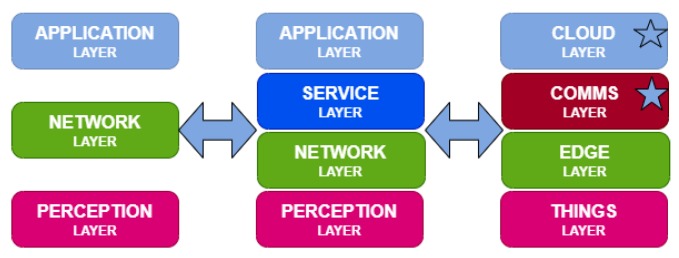
Evolution of the Internet of Things (IoT) architecture for smart agro.

In [4], authors presented a new architecture based on four layers: things, edge, communication, and cloud. Figure 2 shows the architecture models and their evolution. The starred layers are those affected by the information gathered from the nodes.

**Figure 3:**
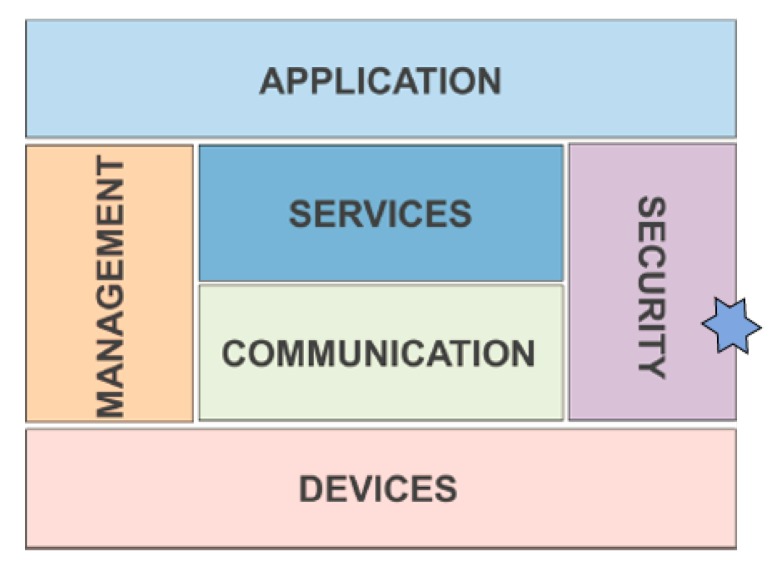
Recommended IoT architecture of smart agro developments.

The recommended architecture for smart-agro IoT platforms, as authors in [2] recommend, is depicted in Figure 3. It is important to note the role of the security layer that connects devices (sensor nodes) with applications (cloud computing, business intelligence layer); therefore, security is an important issue. However, security is not only applied at the highest-level layers but applied from the lower-level layer upwards. The proposed method ensures that the devices are operating correctly, enforcing this layer. The importance of the designed method and the proposed protocol lies in the power to ensure that reliable information will only reach the upper layers (which are precisely the ones that give it the term “smart”-agro). Thus, we are allowing that all the logic associated with crops will not be contaminated with incorrect data, the consequence of which can be fatal for the sustainable aspect of agriculture.

Our proposed method and protocol are both capable of securing the integrity of the sensor nodes, and therefore the quality of the information that reaches the upper layers (responsible for creating intelligence); few resources from these nodes are required to add this extra layer of security that is capable of protecting nodes and information in different stages that last a few milliseconds.

According to the Machina research report, the number of connected agricultural devices is expected to grow to 225 million by 2024. When IoT devices are implemented, the employed communication technologies are a key point to achieve successful operation. Protecting this is necessary to any IoT Smart Agro platform.

The architecture of systems based on IoT and wireless sensor networks is similar to other software architectures or information systems, but with the particularity of remote identification, sensorization, and control of remote objects using devices that embed sensors and actuators [5]. There has been an attempt to standardize the development of IoT-based architectures, but it is still a highly fragmented [6] field, mainly to formalize how to sensor and control objects that provide information, but without success. This seriously affects safety aspects and can compromise the processed information and the strategies derived from this information processing [7]. However, it is worth mentioning that the community is increasingly focused on developing security measures that control the quality of data collected and submited (see Figure 1, as is the case with this paper [8] in which the authors design a data clustering protocol to provide high quality data, it is almost the work presented in [9] that much care is put on the data quality but not on who collects (or injects) it. Our work focuses on the legitimacy of the nodes that provide information. To achieve this, we introduce an implementation of a novel, efficient, and secure protocol to enable the secrecy among the communicating nodes of a WSN based in trusted Key Management sharing scheme to allow the fast rejection of messages or data injected in the network by not authorized sensor nodes. In [10], a classification and overview of the existing protocols is available.

As mentioned above, IoT architectures suffer from significant fragmentation [5]. When their use in the agricultural sector is considered, the issue is even more worrying since the threats to the cyberphysical system can affect not only the infrastructure but also people [11]. The threats that affect these systems are no different from those suffered by any other information system (although the Internet Engineering Task Force has conducted a study on which are the most worrying [12]) and these range from attacks on device firmware to the exploitation of software defects, through device cloning. However, among all the threats that should be prioritized are those that allow an attacker to access the information operating in the sensor network, both to read it and to inject erroneous information that leads to the entire system and its associated processes to states of inoperability and ineffectiveness. Even if the nodes are reverse-engineered, an attacker should not be allowed to inject information into the system, and this is a resolved priority in this work. When the node is manipulated, it is disconnected and the operation key of the rest of the nodes is regenerated.

Our aim in this work is to provide an application of the protocol introduced in [13] for group key management in dynamic communicating groups that behaves very efficiently in the initial key agreement, with just two rounds, and that scales perfectly with just one message per rekeying operation.

## 2. Proposed Cryptographic Methods

It is proposed to create a method to authenticate sensor nodes through which it is possible to identify which nodes will be able to send the information collected from the sensors to the upper layers of the Smart Agro IoT architecture and which nodes will not be able or will simply be ignored. Thus, group-based key distribution methods is a feasible solution to this end. These methods must be carefully designed so that they do not exceed the resources available at the sensor nodes.

Group Key Management (GKM) is a main concern mainly due to the huge development of multiparty communications that apply in many situations nowadays. For this reason, distributed GKM is becoming very popular and there exist many approaches trying to provide effective protocols to this end. In a distributed GKM, members in the group collaborate to build a common key to be used in secure communications. This way, they get somehow federated and a certain level of trust is put in the data they send to the servers. Attacks to these services are quite simple if the sources of the data are not secured. These services are used to implement data mining techniques (Figure 1); therefore, a layer of trust is necessary. An interesting example is that of nodered (http://nodered.org/), a tool for wiring together hardware devices, APIs, and online services that is used in settings like [14]. A tool that by default is not secured; anyone who can access the IP address and port it is running on can access the editor and deploy changes. However, in environments where GKM is used, nodes are securely grouped and the data they send is trusted.

The first key exchange was introduced by Diffie and Hellman in [15] and shows a collaborative way between two communicating parties agreeing in a common way. However, this process between two members in a group does not scale when we are dealing a communication process where members in the group are changing constantly. Thus, many authors try to get extensions of the two-party Diffie–Hellman key exchange that scale for dynamic communication group. One of the widest known works is Cliques, introduced in [16], where the authors provide two different extensions of the Diffie–Hellman key exchange that behave really efficiently in the rekeying process, using just one message to this end. In [17], the authors show a serious security issue for one of the extensions given in [16], unless some authenticating information is used. In [18], the authors provide a second version of the above-mentioned protocols that use a double Diffie–Hellman key exchange to, somehow, authenticate the recovered key, although they cannot avoid every menace that a Public Key Infrastructure does by means of certificates, which are not possible to be used in communication groups such as those given in an ad-hoc network. However, in both approaches, the initial key agreement requires too many rounds with many messages going through the communicating group.

This is also the case of [19], i.e., the initial key agreement does not behave in an efficient way as the number of members increases. A detached approach for the initial key agreement is [20], where the initial key agreement is carried out just in three rounds, independently of the number of members in the group, but rekeying operations reveal not to be as efficient as [16].

### 2.1. The Initial Key Agreement

Let us start by establishing the general setting for the group key management. Participants in the communication process will be given by the set $\left\{U_{1},\ldots U_{n}\right\}$ agreeing on an elliptic curve *E* and a point *P* in *E*, being the set points generated by *P* (possibly the whole set of points of *E*) of prime order and big enough to ensure that the Elliptic Curve Discrete Logarithm Problem is hard to solve. Every participant $U_{i}$ holds two pairs of private-public keys, say $\left(N_{i},N_{i}P\right)$, $\left(X_{i},X_{i}P\right)$. One of these users will be the group controller that we will denote by $U_{c}$, for some *c* in the set {1,…,n} and will be in charge of sending the initial keying information as well as the following rekeying messages. The protocol that describes the initial key agreement is given by the following steps.

*Protocol:*

1. Every user $U_{i}$ publishes the pair $\left(N_{i}P,X_{i}P\right)$, i=1,…,n.
2. The group controller $U_{c}$ broadcasts the keying message
  $${\left\{C_{j}=N_{c}\left(\sum_{r=1,r\neq j,c}^{n}N_{r}\right)P-X_{c}\left(X_{j}P\right)\right\}}_{j=1,j\neq c}^{n}$$
3. Every user $U_{j}$, j=1,…,n j≠c, using his private information, computes
  $$C_{j}+N_{j}\left(N_{c}P\right)+X_{j}\left(X_{c}P\right)$$
4. User $U_{c}$ computes $N_{c}\left(\sum_{r=1,r\neq c}^{n}N_{r}\right)P$

After the precedent protocol, it is clear that all users share the common value $N_{c}\left(\sum_{r=1,r\neq c}^{n}N_{r}\right)P$.

On the other hand, we can observe that the proposed protocol extends the classical Diffie–Hellman key exchange over the group of points in an elliptic curve. To do so, we first note that, given *P* a point in an elliptic curve, it is equivalent to consider a sum of points NP+XP for *N* and *X* random integers and the point (N+X)P. Thus, when n=2, user $U_{1}$ publishes $N_{1}P$ in step 1 and user $U_{c}=U_{2}$ sends $N_{2}P$ and then they compute the common value $(N_{1}N_{2})P$ in steps 3 and 4.

### 2.2. The Rekeying Process

Rekeying is necessary whenever a new member joins the group or someone is leaving, but it may also be needed just due to caducity of the shared key. Let us start by considering this last case.

The process is simple. The group controller $U_{c}$ generates a new integer *Y* and publishes the new pair of public keys ((YN)P,(YX)P). Then, he sends the rekeying message given by

$${\left\{YC_{j}=YN_{c}\left(\sum_{r=1,r\neq j,c}^{n}N_{r}\right)P-YX_{c}\left(X_{j}P\right)\right\}}_{j=1,j\neq c}^{n}$$

and every member recovers the new common key as in step 3 of the protocol.

In case a member leaves the group, say $U_{i}$, then $C_{i}$ is removed and $U_{c}$ sends the rekeying message

$${\left\{YC_{j}\right\}}_{j=1,j\neq c,i}^{n}$$

Finally, in case a new member joins the group, then we can denote him by $U_{n+1}$, who will hold his corresponding two pairs of private-public keys $\left(N_{n+1},N_{n+1}P\right)$, $\left(X_{n+1},X_{n+1}P\right)$ and publish his two public keys. Then, the group controller sends the rekeying message ${\left\{C_{j}^{\prime }\right\}}_{j=1,j\neq c}^{n+1}$ where

$$C_{j}^{\prime }=YC_{j}+YN_{n+1}P,\;j=1,\ldots ,n,\;n\neq c$$

$$C_{n+1}^{\prime }=YN_{c}\left(\sum_{r=1,r\neq c}^{n}N_{r}\right)P-YX_{c}\left(X_{n+1}P\right)$$

and every member recovers the new common value $YN_{c}\left(\sum_{r=1,r\neq c}^{n+1}N_{r}\right)P$ as above, using his private information $\left(N_{i},X_{i}\right)$, i=1,…,n+1, i≠c.

### 2.3. Security Considerations

In the preceding sections, we pointed out that the introduced protocol naturally extends the Diffie–Hellman key exchange over elliptic curves. It is then also natural that, if an attacker is able to solve the Diffie–Hellman Problem, i.e., given the public values $N_{1}P$ and $N_{2}P$, finding $(N_{1}N_{2})P$, then he can get the common key by using $(N_{c}N_{i})P$ and $(X_{c}X_{i})P$ for some i=1,…,n, i≠c. Thus, we should assume that solving this problem is hard.

In case the Diffie–Hellman problem is hard to solve, then a former user could not get values $(YN_{c}N_{i})P$ and $(YX_{c}X_{i})P$ for some i=1,…,n, i≠c to get the new key from the rekeying message after his departure.

A new user $U_{n+1}$ could get $YN_{c}\left(\sum_{r=1,r\neq c}^{n}N_{r}\right)P$ using his private information $X_{n+1}$. However, this does not leak any information on the old key $N_{c}\left(\sum_{r=1,r\neq c}^{n}N_{r}\right)P$ whenever he cannot get *Y* from the pair of (public) points $(YN_{c})P$ and $N_{c}P$ due to the difficulty of solving the Elliptic Curve Discrete Logarithm Problem.

Finally, let us observe the importance of using two pairs of private-public keys. Let us assume that we are using just the pair $\left(N_{i},N_{i}P\right)$, i=1,…,n. Then, the group controller sends the message

$$\left\{C_{j}=N_{c}\left(\sum_{r=1,r\neq j,c}^{n}N_{r}\right)P\right){\}}_{j=1,j\neq c}^{n}$$

If an attacker computes $\sum_{j=1,j\neq c}^{n}C_{j}$, then he gets (n−2)K where $K=N_{c}\left(\sum_{r=1,r\neq c}^{n}N_{r}\right)P$ is the shared key. Now, if the number of elements in the subgroup generated by *P* is a prime *q*, then computing x(n−2)K for x=n−2modq will result in the group key.

Now, we can observe that, using the two pairs of private-public keys, the corresponding sum $\sum_{j=1,j\neq c}^{n}C_{j}$ will give $\left(n-2\right)K-X_{c}\left(\sum_{r=1,r\neq c}^{n}X_{r}\right)P$ that does not reveal anything on the shared key.

## 3. Results: Performance Analysis

The first attempt to create a group key agreement protocol, by means of Diffie–Hellman, is owed to Ingemarsson [21]. This protocol, (cited in this paper as *ING*), has a synchronous startup stage and executes (n−1) rounds. Members are logically connected using a ring topology. Each member, in each round, raises the key received to its exponent and sends this value to the next member.

All the members, after (n−1) rounds, share the key $K_{n}$. A remarkable result, regarding agreement for groups, is owed to Burmester and Desmedt [20]. However, in addition to being efficient, the protocol has been shown to be safe, as long as the Diffie–Hellman problem is safe. Instead, the protocol, named *BD*, is not very suitable for dynamic groups: all members of the group have to refresh their keys to avoid information leakage.

Regarding communication costs (bandwidth), in our case, our protocol is the one that less bandwidth needs as it only requires 1 messages being sent (see Table 1). Another relevant aspect of the efficiency shown by the protocol is the number of messages received and sent per each member since this involves a series of computations that consume time and resources. It is worth noting that messages sent by non-controller users are as small as 2∗size_of_the_datatype. The size of the message sent by the controller is bigger but can be sent efficiently. If we are doing arithmetic with 32 bit-integers, the size of the message sent by the $U_{c}$ needs to be 64∗(2(n−1)) bits.

**Figure 4:**
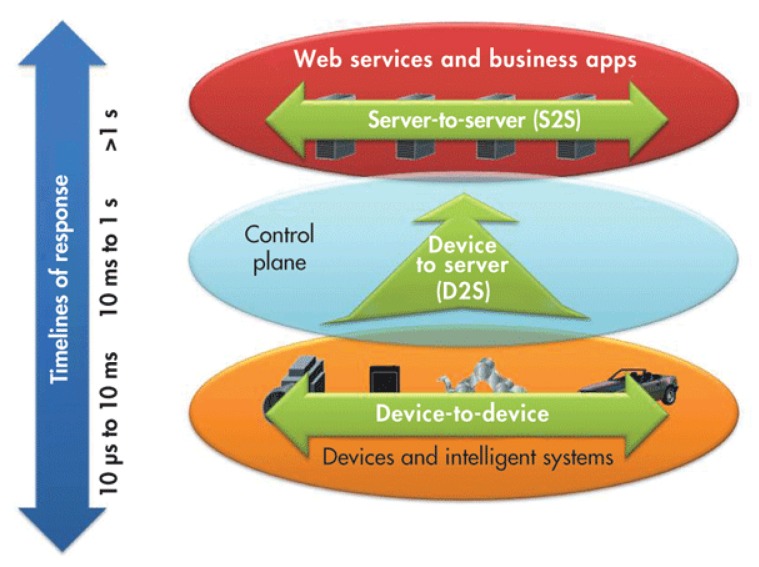
Response times intervals accepted as efficient.

Figure 4 shows that response time of nodes opperating at the sensor network level ranges from 10 µs to 10 ms. It is worth noting that avoiding Modular Exponentiation is important to get all nodes operating fast. Table 1 illustrates clearly that our protocol involves the least overhead with respect to the communication infrastructure: as part of the protocol, each member (node) sends a unique message and receives just one. The protocol is designed to scale well being the unique bottleneck the computation that $U_{c}$ needs to do as the number of users is increased.

If we come to refer again to the efficiency of the protocols, we can mention that the ECC is significantly faster than legacy methods that were based in the exponentiation on a finite field, while providing the same level of security (keeping response times in the ranges depicted in Figure 4). For example, utilizing our protocol, a given security is achieved. For example, with a key size of 160 bits, it is required that the *ING* and *BD* protocols use a key size of 1024 bits to achieve the same level of security. It is worth noting that, although a point on a curve is defined by two coordinates, all elliptic curves are symmetric with respect to the *x*-axis and then only coordinate *x* is represented for each point. Coordinate *y* is calculated rather than stored and an extra bit is added to indicate whether the point in question is on the positive side or the negative side.

### 3.1. Evaluation of the Protocol

**Figure 5:**
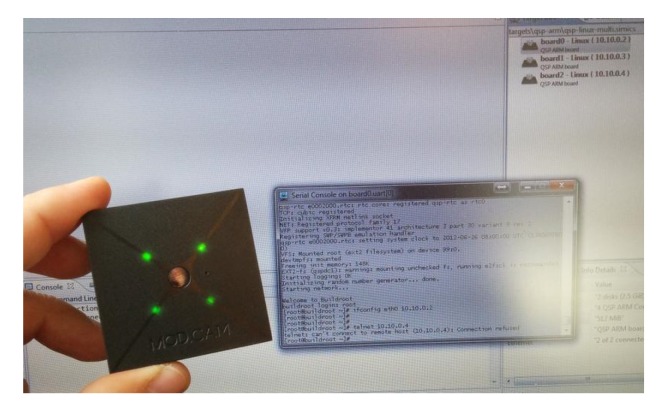
Node of the sensor network.

The sensor nodes as well as the main nodes of the sensor network were run with the configuration of a Minimum Viable Device (MVD), see Figure 5, which is a device that is equipped with, typically, an ARM processor with chipset Qualcomm MSM8974 Snapdragon 800, CPU Quad-core 2.3 GHz Krait 400, GPU Adreno 330 (not used), Storage: 16/32 GB. RAM 2 GB (this is a limiting factor for the $U_{c}$ but we, still, need a high number of nodes to reach it). This sets up the configuration of the physical nodes used for evaluation purposes.

We emulated the mentioned MVD using gem5 [22], which is a cycle-accurate micro-architectural simulator. For micro-architectural simulation with the ARM instruction set architecture (ISA), currently only the out-of-order model (O3) is functional. This model can boot unmodified Linux images in the Full-System (FS) mode, which simulates a complete system.

**Figure 6:**
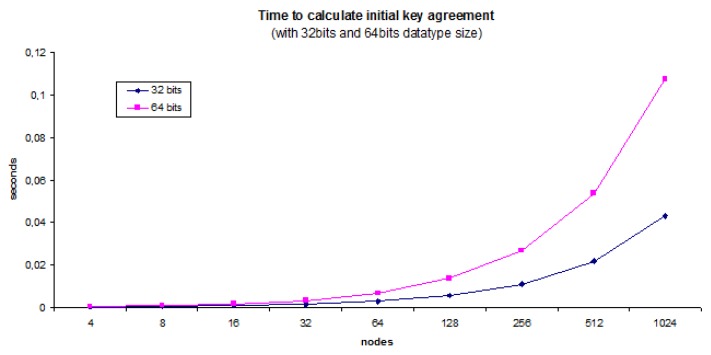
Time $U_{c}$ needs to calculate the initial key agreement message.

There is a clear need to assign the most powerful node as the $U_{c}$ part of the protocol as the computation will be increased with the numbers of users and this affects the rest of the protocol. Users $U_{1}$ to $U_{j}$ except the controller (from i=1,…,n) always operate four modular multiplications and a sum with a number ($C_{j}$) whose size is based on the number of users. Figure 6 shows that node representing $U_{c}$ role in the protocol spends more time in operations when the size of the integers are bigger. We tested native datatypes, with non-native datatypes $U_{c}$ experiencing a bigger delay. In any case, the protocol performs well for a huge number of nodes such as 1024.

**Figure 7:**
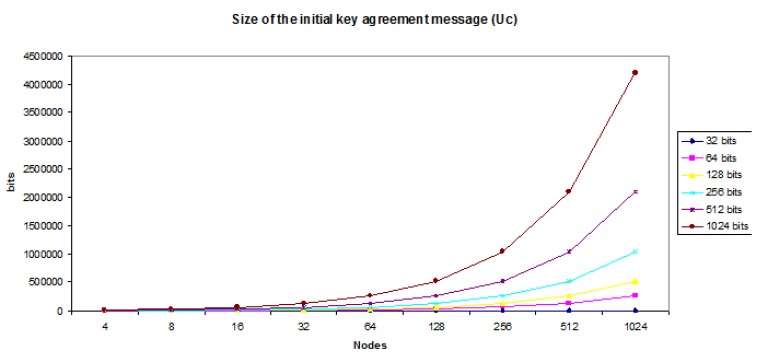
Size of the message created by $U_{c}$.

Figure 7 shows that the real bottleneck is found with the memory needed to hold the message that needs to be sent to the rest of the nodes, as it is a data structure containing a list of items whose size is proportional to the number of participating nodes. Figure 7 shows that even with 1024 nodes and using keys of size 1024, the size of the message is below 1 MB (0.523776 MB).

#### 3.1.1. Testbed for a Physical Node

Despite the fact that the tests carried out in emulated environments involve defining the lower performance level of this proposal, physical nodes have also been included to test the protocol’s efficiency.

The node has been prepared to allow reduced energy consumption due to kernel unnecessary activities. For this purpose, a strategy of modifying the used kernel architecture has been adopted, eliminating unnecessary modules and drivers in a node dedicated to the collection of sensor information and its subsequent sending to higher layer nodes for processing. These nodes, for example, must eliminate everything related to graphic servers, or with policies such as those implemented in udev, sound drivers, etc. All this allows the memory footprint to be less, the activities of the node to be exclusively those that are necessary, avoiding unnecessary energy consumption, or even exposing the node (and the whole infrastructure) to uncontrolled attacks due to vulnerabilities in libraries.

YOCTO has been used to create the dedicated image. Images are created through recipes for embedded systems.The Yocto Project http://www.yoctoproject.org is an open-source industry led consortium that supports Linux-based system platforms for many embedded system applications for a wide variety of processors. The precursor of the yocto project was the OpenEmbedded project http://www.openembedded.org built on the Angstrom distributed kernel.

Table 2 shows data (in milliseconds) for the initial key agreement for a physical node, using 64-bit data types, in a network with 1, 10, 100, and 1000 nodes. Messages latency, below 1 ms, can be ignored in this environment as messages are sent in the WSN environment exclusively (not involving the communication with external nodes -cloud servers, etc.).

#### 3.1.2. Extending the Scalability of the Protocol

**Figure 8:**
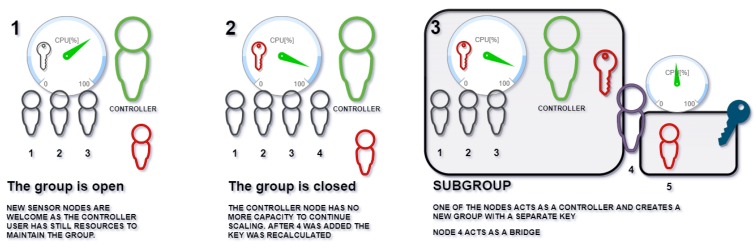
Scaling of the protocol.

Figure 8 shows how the nodes can be reorganized when $U_{c}$ gets overloaded. As the number of members grows, the requirements for each the controller node is increased. Limited capabilities of the controller node set a limit to the number of participants.

Figure 8 shows that, for an efficient number of members (step 1 in Figure 8), adding a new member should be avoided. A node of the group switches its behavior to create an extra subgroup, using a private session key. Node 5 (for this example) is subgrouped to node 3. Node 3 publishes a list of *nodes being supervised*. Node 3 retransmits all the messages received to all the members of its subgroup using a key swap. Messages sent from nodes within the subgroup are forwarded to the general group. Node 3 acts as a proxy (with NAT services). When node 3 leaves, then a message is sent to the subgroup to look for a new subgroup controller.

This approach allows that session keys can be generated faster, as the nodes are segmented. Each segment can operate concurrently. Thus, (i) less memory per node is required, (ii) nodes create groups faster, and (iii) there is extra computation added to each message reception as they must be accessed with a session key and then re-sent with a different session key, but, as the subgroups are not a bottleneck, this operation should not have an effect.

## 4. Conclusions

The infrastructures that support its business intelligence in the generation of strategies based on data processing must not only ensure the processing infrastructure but also ensure that the sensor nodes that monitor fundamental parameters for the crops and its evolution operate reliably and correctly. To ensure the quality of data, there is vast literature, which shows that this is a problem that has generated awareness. However, preventing the identity of a node from being supplanted, or avoiding a node outside the infrastructure to inject data are topics that generate interest in the scientific community but are not yet as well developed.

In this article, we have proposed the design of a distributed and efficient key exchange method as well as the implementation of a protocol based on the key exchange method, using elliptic curve cryptography that provides a solution to security problems in IoT and WSN architectures and frameworks dedicated to precision agriculture and smart-agro. The efficiency and performance against well established protocols in the field of key exchange methods have been demonstrated.

The proposed method can be used in any scenario of the smart agro sector sensor nodes are used to collect different data as pH, temperature, humidity, wind, meteo, etc., which are sent to more complex systems in charge of making decisions based on the processing of the received data. Examples of these decisions are the amount of water to be sent to the plots or to a certain group of olive trees, or some other strategic decisions as time to start harvesting based on the color of the fruit, leaf, etc. Depending on the characteristics of the environment where the implementation of the IoT system is carried out, securing the system may become a challenging task as different types of threats must be considered. Just as an example, in [3], a list of security threats and managing risks, is presented: vulnerable software/code, privacy threats, cloning of things, malicious substitution of things, eavesdropping attacks, man-in-the-middle attack, firmware attacks, extraction of private information, routing attack, elevation of privilege, and denial-of-service (DoS) attacks. Apart from the previously mentioned security threats, physical attacks that the IoT deployment may face are also possible. Our protocol enables the damaged sensor to be aware of the malfunction and request to be excluded from the secure comm group. However, this is possible in case the node belongs to such a group and thus the damaged sensor sends no data to the upper layers. The main aspect is that the IoT devices must be protected. Another threat to the devices deployed on the field is the possibility that they might be replaced with malicious nodes, providing the attacker with access to the network. The deployed devices may also be susceptible to malicious code and false data injection, leading to incorrect results and the malfunctioning of the system. This issue is successfully solved by the protocol, as shown in the mathematical proofs and tests.

The proposed method and protocol can be implemented in devices with severe constraints on computational resources. The cryptography used is supported by elliptic curve cryptography, so the used processors can go from Xmega and the like (used in classic sensor information collection nodes in the agricultural sector) to more advanced processors such as ARMs given that the use of circuitry specially dedicated to cryptography is contemplated.

Memory availability is a carefully designed aspect of the protocol. The greater the available memory, the greater the key used and/or the greater number of nodes that can be part of the secured network. However, efforts have been made to interfere as little as possible in the memory footprint in order to leave intact the operation of the data collection and sensor control applications.

The energy consumption is negligible, especially when compared to the node’s own operations: collecting data from the sensors, preparing the data from the sensors, checking the operating ranges and sending the information to the collecting node, not only because of the type of computational tasks but because of the frequency with which they are performed.

The energy consumption is negligible, especially when compared to the node’s own operations: collecting data from the sensors, preparing the data from the sensors, checking the operating ranges and sending the information to the collecting node—not only because of the type of computational tasks but because of the frequency with which they are performed. By using the emulator, we wanted to define a lower performance bound for the protocol, independent from the myriad of architectures available in the market. However, on the ARM device, the rekeying stage lasts no longer than 0.5 to 2 ms depending on the participating nodes.

An interesting aspect regarding embedded systems (important part of Internet of Things) is the restricted operating environment they are tied to (computing power, power source, …). In this paper, it has been shown that the method and protocol are highly scalable for group key agreement. The agreement is made using multicast messages. The proposal devised reduces the time of operation for the key agreement and the memory footprint. In addition, an alternative method to avoid potential bottlenecks, when the number of sensor nodes grows rapidly, was also sketched. This method is based on subgrouping (or segmenting) the network. New members can join these segments. The host node acts as a bridge between group and subgroup by means of a small process we created.

## Figures and Tables

**Table 1:** Comparison of protocols.

	Protocol	ING	BD	Ours
Parameter				
Rounds		n−1	2	2
# messages		n(n−1)	2n	(n−1)
				1 by $U_{c}$
Msgs sent		n−1	2	1
				1 $U_{c}$
Msgs received		n−1	n+1	1
Modular Exp. by $U_{i}$		*n*	n+1	-
Modular Mult. by $U_{i}$		-	-	4 by $U_{i}$
				2(n−1)+1 $U_{c}$

**Table 2:** Time (in ms) to get a node fully available (initial key agreement) in the secured wireless sensor network (WSN), using keys of 1024 bits.

1 Node	10 Nodes	100 Nodes	1000 Nodes
0.42	0.49	0.78	1.91

## Abbreviations

The following abbreviations are used in this manuscript:

IoT	Internet of Things.
MVD	Minimum Viable Device.
WSN	Wireless Sensor Network.
GKM	Group Key Management.
API	Application Programming Interface.
ECC	Elliptic Curve Cryptography.
ISA	Instruction Set Architecture.
FS	Full System.
